# Alcohol, DNA Methylation, and Cancer

**DOI:** 10.35946/arcr.v35.1.04

**Published:** 2013

**Authors:** Marta Varela-Rey, Ashwin Woodhoo, Maria-Luz Martinez-Chantar, José M. Mato, Shelly C. Lu

**Affiliations:** **Marta Varela-Rey, Ph.D.**, *is a CIBERehd postdoctoral fellow, at the Centro de Investigación Cooperative en Biosciencias (CIC bioGUNE), Centro de Investigación Biomédica en Red de Enfermedades Hepáticas y Digestivas (CIBERehd), Technology Park of Bizkaia, Derio, Spain.*; **Ashwin Woodhoo, Ph.D.**, *is a Ramón y Cajal postdoctoral fellow, at the Centro de Investigación Cooperative en Biosciencias (CIC bioGUNE), Centro de Investigación Biomédica en Red de Enfermedades Hepáticas y Digestivas (CIBERehd), Technology Park of Bizkaia, Derio, Spain.*; **Maria-Luz Martinez-Chantar, Ph.D.**, *is group leader, at the Centro de Investigación Cooperative en Biosciencias (CIC bioGUNE), Centro de Investigación Biomédica en Red de Enfermedades Hepáticas y Digestivas (CIBERehd), Technology Park of Bizkaia, Derio, Spain.*; **José M. Mato, Ph.D.**, *is director of CIC bioGUNE and of the Center for Cooperative Research in Biomaterials (CIC bioMAGUNE), as well as research professor and group leader at CIC bioGUNE, (CIBERehd), Technology Park of Bizkaia, Derio, Spain.*; **Shelly C. Lu, M.D.**, *is professor of medicine, associate chief of the Division of Gastrointestinal and Liver Diseases, and associate director of the USC Research Center for Liver Diseases, Keck School of Medicine, University of Southern California, Los Angeles, California.*

**Keywords:** Alcohol consumption, chronic alcohol use, alcohol use disorders, alcohol-induced cancer development, risk factors, epigenetics, epigenetic mechanisms, cancer, upper aerodigestive tract cancer, colorectum cancer, breast cancer, carcinogenesis, DNA methylation

## Abstract

Cancer is one of the most significant diseases associated with chronic alcohol consumption, and chronic drinking is a strong risk factor for cancer, particularly of the upper aerodigestive tract, liver, colorectum, and breast. Several factors contribute to alcohol-induced cancer development (i.e., carcinogenesis), including the actions of acetaldehyde, the first and primary metabolite of ethanol, and oxidative stress. However, increasing evidence suggests that aberrant patterns of DNA methylation, an important epigenetic mechanism of transcriptional control, also could be part of the pathogenetic mechanisms that lead to alcohol-induced cancer development. The effects of alcohol on global and local DNA methylation patterns likely are mediated by its ability to interfere with the availability of the principal biological methyl donor, S-adenosylmethionine (SAMe), as well as pathways related to it. Several mechanisms may mediate the effects of alcohol on DNA methylation, including reduced folate levels and inhibition of key enzymes in one-carbon metabolism that ultimately lead to lower SAMe levels, as well as inhibition of activity and expression of enzymes involved in DNA methylation (i.e., DNA methyltransferases). Finally, variations (i.e., polymorphisms) of several genes involved in one-carbon metabolism also modulate the risk of alcohol-associated carcinogenesis.

According to the World Health Organization (WHO) Global Burden of Disease Project, alcohol of all deaths per year worldwide (corresponding to 1.8 million people) and is causally related to more than 60 different medical conditions (Rehm et al. 2004). Cancer formation (i.e., carcinogenesis) is one of the most significant consequences attributed to alcohol consumption, and approximately 3.6 percent of all cancer-related cases (5.2 percent in men and 1.7 percent in women) worldwide, as well as 3.5 percent of all cancer-related deaths are related to chronic alcohol drinking (Boffetta et al. 2006). Based on available epidemiological data, an international group of epidemiologists and alcohol researchers concluded that alcohol induces carcinogenesis in numerous organs, including the upper aerodigestive tract, liver, colorectum, and female breast (Baan et al. 2007).

Several pathogenic mechanisms contribute to alcohol-induced carcinogenesis in each type of cancer. The most commonly cited mechanisms include the effect of acetaldehyde—the first metabolite of ethanol oxidation—and oxidative stress (Seitz and Stickel 2007). Increasing evidence shows that alcohol also can induce epigenetic alterations, for example, in pathological conditions such as fetal alcohol spectrum disorders (Kobor and Weinberg 2011). Epigenetic alterations also are a hallmark of cancer development in general (Esteller 2008). Therefore, this article reviews the available evidence that such changes also could be an important contributory factor to alcohol-induced carcinogenesis. In particular, it discusses the role of DNA methylation in carcinogenesis and how alcohol may affect the pathways that regulate the availability of S-adenosylmethionine (SAMe), the principal biological methyl donor for methylation reactions. (For a list of abbreviations of the names for genes, proteins, and other compounds as well as their functions, see table).

## DNA Methylation and Cancer

**Figure 1:**
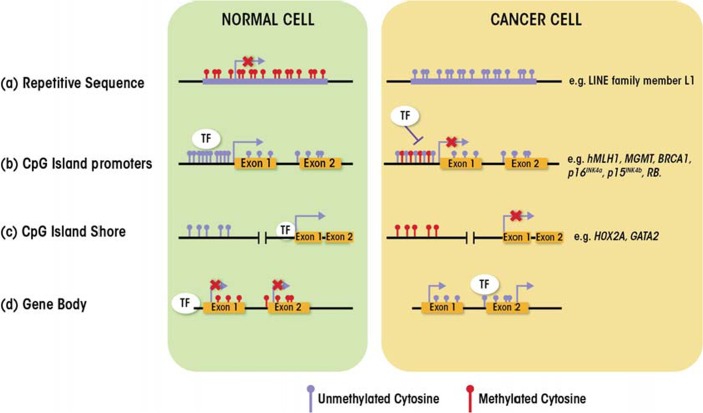
DNA methylation patterns in normal and cancer cells. (a) Repetitive sequences generally are methylated at cytosine nucleotides in normal cells. Global loss of methylation in cancer cells leads to chromosomal instability and activation of endoparasitic sequences. (b) CpG islands in promoter sequences typically are unmethylated in normal cells whereas they can become hypermethylated in cancer cells, leading to transcriptional repression. Examples of genes affected are shown on the right. (c) Similar patterns are seen in CpG island shores, located in front (i.e., upstream) of promoters. (d) CpGs located in gene bodies frequently are methylated in normal cells; this pattern is reversed in cancer cells, leading to initiation of transcription at several incorrect sites.

DNA methylation plays a critical role in the control of gene activity. This methylation almost exclusively involves the addition of a methyl group to carbon 5 of cytosine nucleotides, and specifically those cytosines that precede guanines (i.e., are part of CpG dinucleotides). CpG dinucleotides tend to cluster either in regions called CpG islands, which are located in approximately 60 percent of human gene promoters, or in regions that contain large repetitive DNA sequences (e.g., centromeres and retrotransposons). In the former case, the CpG dinucleotides generally tend to remain unmethylated, whereas in the latter case they mostly are methylated to prevent chromosome instability (Rodriguez-Paredes and Esteller 2011) (figure 1). DNA methylation also occurs in CpG island shores—that is, regions of lower CpG density that are located close to CpG islands (i.e., within 2 kb). In these three cases, DNA methylation typically is associated with repression of gene expression (i.e., transcription). In a few cases, however, DNA methylation also can enable gene transcription, namely when the methylation occurs in the body (i.e., coding sequences) of the gene rather than the promoter (Portela and Esteller 2010).

DNA methylation is mediated (i.e., catalyzed) by three main enzymes called DNA methyltransferases (DNMTs), all of which transfer a methyl group from SAMe to DNA:

- DNMT1, which also is referred to as the maintenance DNMT, completes the methylation of the partially methylated (i.e., hemimethylated) DNA that is present in the cell after cell division and the accompanying DNA replication.
- DNMT 3A and DNMT 3B are referred to as de novo methyltransferases because they target unmethylated CpGs.

Aberrant epigenetic regulation, including altered DNA methylation, characterizes a wide range of diseases, including cancer (Portela and Esteller 2010). Compared with normal cells, the epigenome of cancer cells shows profound changes in DNA methylation patterns as well as histone modifications patterns (Rodriguez-Paredes and Esteller 2011), including the following:

- Genome-wide hypomethylation (about 20 to 60 percent less overall 5-methyl-cytosine compared with normal cells); global loss of DNA methylation occurs at many genomic regions, such as repetitive elements and retrotranposons, resulting in chromosomal instability, and activation of transposable elements and endoparasitic sequences (e.g., LINE family member L1) (figure 1).
- Hypomethylation at specific promoters, resulting in aberrant activation of cancer-inducing genes (i.e., oncogenes) (e.g., *SERPINB5*) and inducing loss of imprinting at other DNA sites (e.g., *IGF2*).
- Hypermethylation of CpG island promoters of genes involved in central cellular pathways, such as DNA repair (e.g., at genes called *hMLH1, MGMT*, and *BRCA1*) and cell cycle control (e.g., at genes called *p16INK4a, p15INK4b*, and *RB*), resulting in the silencing by is the most recognized epigenetic disruption in human tumors.
- Aberrant DNA methylation at CpG island shores (e.g., in genes called *HOX2A* and *GATA2*) (Portela and Esteller 2010; Rodriguez-Paredes and Esteller 2011).

## One-Carbon Metabolism

**Figure 2:**
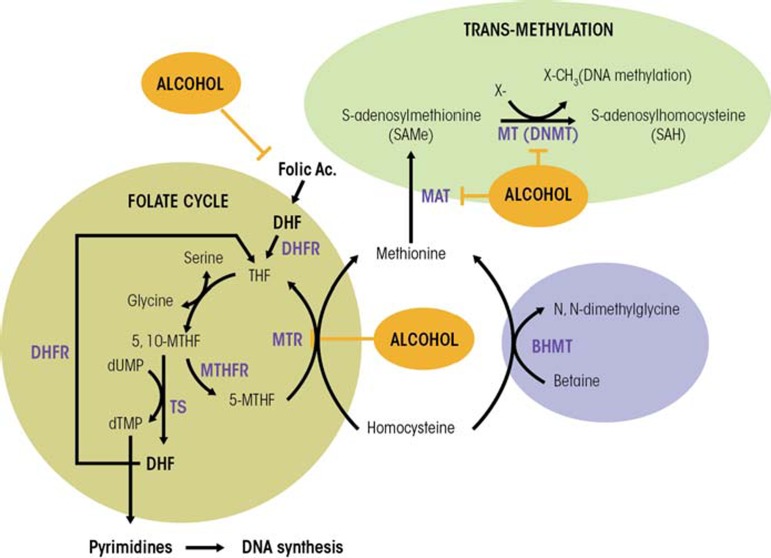
One-carbon metabolism with a schematic representation of the role of methionine in folate metabolism and transmethylation reactions and steps that are inhibited by alcohol. BHMT: betaine homocysteine methyltransferase; DHF: dihydrofolate; DHFR: dihydrofolate reductase; DNMT: DNA methyltransferase; dTMP: deoxythymidine monophosphate; dUMP: deoxyuridine monophosphate; Hcy: homocysteine; MAT: methionine adenosyl transferase; Met: methionine; MT: methyltransferase; 5-MTHF: 5-methyltetrahydrofolate; 5,10-MTHF: 5,10-methylenete-trahydrofolate; MTHFR: methylenetetrahydrofolate reductase; MTR: methionine synthase; SAH: S-adenosylhomocysteine; SAMe: S-adenosyl-methionine; THF: tetrahydrofolate; TS: thymidylate synthase.

Persuasive evidence indicates that several dietary factors, such as alcohol, can modulate DNA methylation patterns and increase susceptibility to disease, including cancer, by altering one-carbon metabolism (Choi and Friso 2010). The term one-carbon metabolism refers to a network of biochemical reactions in which a chemical unit containing one carbon atom (e.g., a methyl group) is transferred through several steps from a donor to another compound, such as DNA (figure 2). The first step in this chain of events is the transfer of the one-carbon group to tetrahydrofolate (THF), resulting in the formation of 5,10-MTHF. This molecule then can be used in the synthesis of the nucleotide thymidine in a reaction catalyzed by the enzyme thymidylate synthase (TS). In this reaction, one carbon group of the 5,10-MTHF is transferred to a molecule called deoxyuridine monophosphate (dUMP), resulting in the formation of deoxythymidine monophosphate (dTMP) and dihydrofolate (DHF). This reaction is considered to be a limiting step in DNA synthesis and, more importantly, reduces dUMP levels, which can lead to breaks in both strands of the DNA molecules. Finally, the enzyme dihydrofolate reductase (DHFR) catalyzes the reduction of DHF back into THF (Mato et al. 2008; Van der Put et al. 2001).

In an alternative set of reactions, 5,10-MTHF also can be converted to 5-methyltetrahydrofolate (5-MTHF) in a reaction catalyzed by the enzyme methylenetetrahydrofolate reductase (MTHFR). The 5-MTHF generated then can be used in the remethylation of homocysteine (Hcy) to methionine, which is catalyzed by methionine synthase (MTR). In the liver and kidney, Hcy also can be remethylated into methionine through a folate-independent reaction, catalyzed by betaine homocysteine methyltransferase (BHMT). BHMT requires betaine, a break-down product (i.e., metabolite) of choline for its activity.

Methionine, in turn, is an essential amino acid that, together with ATP, participates in the formation of SAMe, in a reaction catalyzed by methionine adenosyltransferase (MAT). SAMe is the principal methyl donor for numerous reactions, including protein, RNA, DNA, and histone methylation. The formation of SAMe is a limiting step in DNA methylation. After transfer of its activated methyl group in the methylation reactions, SAMe is converted into S-adenosylhomocysteine (SAH). This compound also can affect DNA methylation because it is a potent competitive inhibitor of methyltransferases, including DNMTs. Both an increase in SAH levels and a decrease in the SAMe-to-SAH ratio can inhibit transmethylation reactions (Lu 2000).

### Polymorphisms of One-Carbon Metabolism Enzymes

Several studies have shown that variations (i.e., polymorphisms) in several of the genes encoding enzymes involved in one-carbon metabolism and, consequently, in the resulting enzymes can impact the levels of various metabolites generated during these reactions. For some of these metabolites, altered levels in the body can be associated with either increased or decreased cancer susceptibility.

#### Polymorphisms in the Gene Encoding MTHFR

As mentioned above, MTHFR plays a central role in methionine formation by mediating the synthesis of 5-MTHF, which serves as a substrate for the remethylation of Hcy to methionine. Several polymorphisms in the *MTHFR* gene have been reported; two of these, known as the *C677T*^1^ and *A1298C MTHFR* variants, in particular have been investigated in relation to cancer susceptibility. The *C677T* variant occurs in 8 to 15 percent of the population and results in a reduction of *MTHFR* activity. Thus, compared with people carrying two copies of the normal *MTHFR* gene (i.e., homozygous for *MTHFR C* [*CC*]), people carrying one normal gene copy and one variant gene copy (i.e., heterozygous with one *MTHFR C* and one *MTHFR T* [*CT*]) show a 45 percent reduction in *MTHFR* activity, and those homozygous for the *MTHFR T* variant (*TT*) show a 70 percent decrease of *MTHFR* activity (Motulsky 1996; Van der Put et al. 1995). Persons with the TT genotype have low levels of folate and vitamin B12 in the blood, as well as increased Hcy levels. Studies of the plasma folate and Hcy levels in people carrying one or two copies of the *A1298C MTHFR* polymorphism, however, have yielded inconsistent results (Sharp and Little 2004).

#### Polymorphisms in the Gene Encoding TS

TS catalyzes the conversion of dUMP into dTMP using 5,10-MTHF. Because dUMP can be misincorporated into new DNA strands during DNA replication, which can cause breaks in the double-stranded DNA molecules, the actions of TS can reduce the occurrence of DNA double-strand breaks. Two polymorphisms in the *TS* gene may be associated with an increased cancer risk:

- The presence of a repeated sequence of 28 nucleotides (i.e., a 28-bp tandem repeat) at the beginning of the *TS* gene (i.e., in the *TS* 5′-untranslated enhanced region); this variant is referred to as *TSER*;
- A 6-bp deletion/insertion at the end of the *TS* gene (i.e., in the *TS* 3′-untranslated region); this variant is known as *TS1494del6*.

#### Polymorphisms in the Gene Encoding MTR

MTR catalyzes the remethylation of Hcy to methionine; its activity is essential to maintain adequate folate pools. Most studies suggest that plasma Hcy levels are lower in carriers of a variant of the MTR gene called A2756G. To function properly, MTR must be maintained in an active form; this is achieved by another enzyme called MTR reductase (MTRR), for which polymorphisms also exist. For example, people homozygous for the A66G MTRR polymorphism had elevated levels of Hcy, indicating that the resulting MTRR enzyme led to higher-than-normal MTR activity (Van der Put et al. 2001).

## Alcohol and DNA Methylation

As mentioned above, several pathogenetic mechanisms for alcohol-induced carcinogenesis have been described, including the effects of acetaldehyde and oxidative stress (Seitz and Stickel 2007). More recently, increasing evidence indicates that alcohol may induce epigenetic alterations, in particular aberrant DNA methylation patterns, which also could be important contributory factors to alcohol-induced carcinogenesis. For example, excessive alcohol use is associated with increased risk of colon cancer, which is characterized by global DNA hypomethylation as well as hypermethylation of certain genes (see below). Several mechanisms have been described to date that could contribute to these DNA methylation changes in cancer. These generally are associated with modulation of the pathways that regulate the availability of SAMe.

### Alcohol and Lipotropes

The term lipotropes denotes compounds that help catalyze the breakdown of fat molecules in the body. Lipotropic nutrients (e.g., methionine, choline, folate, and betaine) are important dietary methyl donors and cofactors that play key roles in one-carbon metabolism. Dietary lipotropes influence the availability of SAMe and, consequently, may influence genomic DNA methylation patterns and the expression of multiple cancer-related genes. For example, methyl-deficient diets can induce the development of liver tumors (i.e., hepatocarcinogenesis) in rats by causing global and gene-specific hypomethylation (for a review, see Ross and Poirier 2002). Chronic alcoholics frequently suffer from malnutrition that results in depletion of lipotropes (Seitz and Stickel 2007). The lack of these nutrients in heavy drinkers could possibly result in an altered SAMe production, leading to changes in DNA methylation.

Similarly, malnutrition in alcoholics leads to a severe deficiency of other cofactors of one-carbon metabolism, such as folate (see below), vitamin B6, and vitamin B12. Long-term dietary intake of vitamin B6 is inversely correlated with the risk of developing colorectal cancer in women—that is, inadequate vitamin B6 intake increases the risk of this cancer. This effect is aggravated by chronic consumption of alcohol (Larsson et al. 2005).

### Alcohol and Folate Status

Epidemiological studies have suggested that reduced folate levels in the body increase the risk of several types of cancer, including those of the upper aerodigestive tract, colon/rectum, and breast (Kim 2005). For example, heavy drinkers with low methionine and folate levels have a significantly increased relative risk (RR) for colorectal cancer compared with occasional drinkers with normal methionine and folate intake (Giovannucci et al. 1995). In chronic alcoholics, serum folate levels are significantly reduced compared with healthy subjects (Cravo et al. 1996), likely because folate absorption is reduced in these patients (Halsted et al. 1971). Polymorphisms of the *MTHFR* genes also lead to reduced folate levels, but their contribution to carcinogenesis is tissue-dependent and often contradictory. This is discussed in more detail below.

Two main mechanisms have been described that may explain the cancer-promoting effects of limited folate levels: increased DNA instability and aberrant DNA methylation patterns (Hamid 2012). Folate deficiency alters the balance of the pool of nucleotides needed for the synthesis of new DNA molecules, leading to dUMP accumulation. As a result, dUMP is misincorporated into new DNA molecules; this and the subsequent repair processes can lead to double-strand breaks in the DNA and chromosomal damage, ultimately resulting in cancer. The aberrant DNA methylation patterns associated with folate deficiency are the result of folate’s role in one-carbon metabolism (see figure 2). As mentioned above, 5-MTHF can be used in the remethylation of Hcy to methionine, which in turn generates SAMe. Many studies have shown that folate deficiency reduces SAMe levels and the SAMe-to-SAH ratio as well as increases SAH concentrations (Kim 2005), all of which might contribute to carcinogenesis.

### Alcohol and MTR Activity

Alcohol also can affect SAMe, SAH, and Hcy levels by reducing MTR activity, which in turn results in decreased SAMe levels and enhanced generation of Hcy and SAH (Barak et al. 1996) (see figure 2). To compensate for this decrease in MTR activity, the activity of BHMT is induced after alcohol ingestion. However, after extended periods of alcohol exposure this alternate pathway cannot be maintained. This results in a decrease in the hepatocyte level of SAMe, increases in SAH and Hcy levels, and a reduced SAMe-to-SAH ratio. These effects may contribute to the reduced hepatic SAMe levels observed in patients hospitalized for alcoholic hepatitis (Lee et al. 2004).

### Alcohol and MAT Activity

As mentioned earlier, alcohol also contributes to the generation of oxidative stress through various mechanisms (for a review, see Seitz and Stickel 2007). Increased oxidative stress, in turn, can, at least in the liver, inactivate the MAT I/III enzymes that convert methionine to SAMe (see figure 2). This inactivation results from the covalent modification of a critical cysteine residue at position 121 by nitric oxide and hydroxyl radicals. MAT inhibition causes decreased SAMe levels, leading to reduced methylation reactions. The relevance of this pathway was demonstrated by findings that patients with alcoholic cirrhosis exhibit decreased hepatic MAT activity and SAMe formation (Lu and Mato 2008). In addition, patients with alcoholic hepatitis also show reduced expression of the *MAT1A* gene that encodes the MAT isoenzymes in normal liver, thereby contributing to lower hepatic SAMe levels (Lee et al. 2004).

### Alcohol and DNMT Activity

Finally, both ethanol and its first breakdown product (i.e., acetaldehyde) can impact methylation patterns by altering DNMT activity. Thus, studies found that acetaldehyde can inhibit DNMT activity in vitro (Garro et al. 1991) and that alcohol reduced *DNMT* mRNA levels in rats treated with alcohol for 9 weeks (Bielawski et al. 2002). Similarly, studies in humans found that *DNMT3a* and *DNMT3b* mRNA levels were significantly reduced in patients with chronic alcoholism compared with healthy control subjects (Bonsch et al. 2006).

## Alcohol, DNA Methylation, and Cancer

As described above, alcohol can interfere with one-carbon metabolism in several ways, thereby potentially generating aberrant DNA methylation patterns. The following sections review the currently available evidence indicating that alcohol-mediated changes in DNA methylation profiles contribute to the four main alcohol-associated cancers.

### Liver Cancer

Liver cancer (i.e., hepatocellular carcinoma [HCC]) is a major cause of cancer-related death worldwide. The major risk factors for developing HCC are viral infection (i.e., with the hepatitis B or C viruses), chronic alcoholism, and exposure to toxic substances called aflatoxins. Alcohol remains the major cause of liver-related disease and deaths in the United States (Moghe et al. 2011). Alcohol-induced aberrant DNA methylation has been well characterized as a pathogenetic mechanism contributing to liver disease. Significant global DNA hypomethylation is associated with HCC with several etiologies, including chronic alcoholism, which may result in malignant transformation through mechanisms such as loss of imprinting and chromosomal instability, as described above (Calvisi et al. 2007; Hernandez-Vargas et al. 2010). Similar to other types of cancer, global DNA hypomethylation in HCC is accompanied by greater-than-normal methylation levels (i.e., hypermethylation) at certain CpG sites. For example, a study analyzing 1,505 CpG sites in the promoter regions of 807 cancer-related genes in HCC tissues with different underlying causes, including chronic alcohol consumption, demonstrated an altered methylation pattern in 94 genes. Furthermore, for specific subsets of genes significant associations existed between methylation patterns and tumor progression (i.e., stage of the tumor and grade of differentiation) and background (i.e., cirrhotic versus non-cirrhotic surrounding tissue) (Hernandez-Vargas et al. 2010). Genes exhibiting hypermethylation included *RASSF1, APC*, and *CDKN2A*, all of which have important functions in the liver (Tischoff and Tannapfel 2008). A more recent study (Lampert et al. 2011) also detected aberrant hypermethylation of several genes, including *RASSF1, GSTP1, MGMT*, and *CHRNA3*, in alcohol-associated HCC. Finally, mRNA expression of RB1, an important cell cycle regulator, is decreased as a result of promoter methylation (Edamoto et al. 2003).

The link between alcohol and the mechanisms leading to aberrant methylation has been well elucidated in HCC. Chronic alcoholic patients have reduced hepatic MAT activity resulting from both decreased expression of the *MAT1A* gene and inactivation of the MAT I/III proteins. Reduced MAT activity, in turn, leads to decreased SAMe biosynthesis (Tsukamoto and Lu 2001), which may contribute to the severe loss of global DNA methylation in HCC. Similarly, micropigs fed ethanol show reduced hepatic MTR activity and SAMe/SAH ratio (Halsted et al. 1996) and rats fed ethanol exhibit decreased SAMe levels and global DNA hypomethylation.

Patients heterozygous or homozygous for the previously mentioned C677T *MTHFR* polymorphism have been shown to have a lower risk of developing alcohol-related HCC, but not of HCC with other etiologies (Saffroy et al. 2004). Similarly, people carrying variant alleles of both *MTHFR* and *TS* genes had a statistically significant reduced risk of developing HCC (Yuan et al. 2007). However, these findings sharply contradict another study showing that male patients with alcoholic cirrhosis who were homozygous for the *C677T MTHFR* polymorphism had an increased risk of developing HCC (Fabris et al. 2009). How can these conflicting reports be explained? In principle, the *C677T MTHFR* polymorphism has both protective and cancer-promoting effects. On the one hand, it can have a protective effect because it leads to an increase in the supply of 5,10-MTHF, which can serve as a substrate in the conversion of dUMP to dTMP. This would reduce misincorporation of dUMP into DNA, preventing DNA double-strand breaks and chromosomal instability. On the other hand, the reduced activity of the MTHFR enzyme encoded by the *C677T MTHFR* polymorphism would lead to lower levels of 5-MTHF that is used for the remethylation of Hcy to methionine, which in turn generates SAMe. Thus, the polymorphism would result in lower SAMe levels and SAMe/SAH ratio as well as increased SAH concentrations, which would then contribute to carcinogenesis.

The overall effects of the *C677T MTHFR* polymorphism can be determined when folate levels are taken into account. In situations of adequate folate supply, the levels of 5,10-MTHF increase, leading to a protective effect as described above, possibly counteracting the effects of reduced SAMe. In folate-deficiency situations, however, the levels of both 5,10-MTHF and SAMe would be reduced, leading to chromosomal instability that would be exacerbated by DNA hypomethylation induced by reduced SAMe. This model is consistent with a study in colon cancer showing that patients with the CC genotype have significantly reduced risk of cancer development in situations of adequate folate supply, but that this protection is absent in folate deficiency (Ma et al. 1997). It also can reconcile the contradictory findings of the two studies mentioned above. In the study by Saffron and colleagues (2004), folate levels were similar between healthy and alcoholic patients, thus favoring the protective role of the polymorphism. In the study by Fabris and colleagues (2009), conversely, increased risk of HCC in people with the polymorphisms was only found in males, and not in females. Although the investigators did not measure folate levels, it is plausible that these levels would be lower in men, who tend to be heavier and more regular drinkers compared with women.

### Colorectal Cancer

Colorectal cancer is the third most common cancer and the second leading cause of cancer deaths for both sexes (Marin et al. 2012). Alcohol is a likely etiologic factor for this cancer (Baan et al. 2007). Several studies have indicated that epigenetic processes play a role in alcohol-related colorectal carcinogenesis. For example, rats chronically fed alcohol show genomic DNA hypomethylation but a normal pattern of methylation of the gene *TRP53*, which encodes a protein called p53, in the colonic mucosa (Choi et al. 1999). In a cohort of 609 patients, excessive alcohol use was associated with increased risk of colon cancer with global DNA hypomethylation (Schernhammer et al. 2010). In addition, people with low folate intake/high alcohol intake show a higher frequency of promoter methylation of genes involved in colorectal cancer carcinogenesis (e.g., *APC-1A, p14ARF, p16INK4a, hMLH1, O6-MGMT*, and *RASSF1A*) compared with people with high folate intake/low alcohol intake (Van Engeland et al. 2003). Many of these genes have fundamental roles in many cellular pathways, including DNA repair (e.g., *hMLH1, O6-MGMT*) and cell cycle control (e.g., *p16INK4a*) (Portela and Esteller 2010).

The association between *MTHFR* polymorphisms and colon cancer has been studied extensively, and several factors have been found to influence the relation between the *MTHFR* variants and cancer risk (for a review, see Sharp and Little 2004). For example, one study (Iacopetta et al. 2009) has shown that colon cancer risk in homozygous carriers was dependent on the location of the cancer in the colon. The investigators observed that the TT genotype was associated with an increased risk for of cancer in colon regions closer to the small intestine (i.e., proximal colon cancer) (adjusted odds ratio (AOR) = 1.29) but with a decreased risk for cancer in colon regions closer to the rectum (i.e., distal cancers) (AOR = 0.87). More importantly, the increased risk for proximal cancers was especially pronounced in individuals with high alcohol consumption (AOR = 1.90). Microsatellite instability also has been related with colon cancer risk in people with the TT genotype (Shannon et al. 2002). Finally, the dietary intake of folate and alcohol also has been associated with colon cancer risk in people carrying the *MTHFR* C variant. Thus, Kim and colleagues (2012) observed that low folate intake together with high alcohol intake increased the risk of colon cancer in people with either the CC or the CT genotype.

A subset of colorectal cancers exhibit promoter methylation in multiple genes; these tumors are referred to as the CpG island methylator phenotype (CIMP) (Toyota et al. 1999). The frequency of these tumors depends on the location of the cancer. Thus, 30 to 40 percent of sporadic proximal colon cancers are CIMP^+^, compared with 3 to 12 percent of distal colon and rectal cancers (Curtin et al. 2011). The presence of the A1298C *MTHFR* polymorphism, interacting with diet, may be involved in the development of highly CpG-methylated colon cancers. Homozygous and heterozygous genotypes in conjunction with a high-risk dietary pattern (i.e., low folate and methionine intake and high alcohol use) were associated with CIMP^+^ phenotype (Curtin et al. 2007).

### Breast Cancer

Breast cancer is the second leading cause of cancer death among women (DeSantis et al. 2011). Low doses of alcohol consumption (i.e., ≤1 drink/day) increase the risk of breast cancer by about 4 percent (Hamajima et al. 2002), whereas heavy alcohol consumption (i.e., ≥ 3 drink/day) is associated with an increase in risk of 40 to 50 percent (Pelucchi et al. 2011; Seitz et al. 2012). In addition, high frequency of alcohol consumption is associated with increased breast cancer mortality (Allemani et al. 2011). The role of epigenetic mechanisms in alcohol-related breast cancer also has been investigated. In a recent study of the methylation profiles of 1,413 CpG sites, Christensen and colleagues (2010) showed a strong trend toward decreased DNA methylation with increasing alcohol intake, and a trend toward increased methylation with increasing dietary folate. Other studies have shown altered methylation patterns for several genes associated with alcohol consumption, including hyper-methylation of the *ER-a* (Zhu et al. 2003) and *E-cadherin* genes (Tao et al. 2011) and hypomethylation of *p16* (Tao et al. 2011).

As with other cancers, the person’s genotype for the *C677T MTHFR* variant modulates the effect of alcohol consumption on breast cancer risk. Thus, women with the TT genotype are at a higher risk of breast cancer than those with other genotypes. In postmenopausal women, the breast cancer risk was increased in women with the *C677T MTHFR* variant who had high lifetime daily alcohol intake, suggesting that folate metabolism has an impact on cancer development (Platek et al. 2009). As mentioned earlier, chronic alcohol abuse can cause folate deficiency, which is a well-documented risk factor for breast cancer (Sellers et al. 2001). Why this risk is only observed in post-menopausal women is not clear, but the levels of estrogen in the woman’s body may play a role. Alcohol can interfere with estrogen pathways and increase the levels of estrogen in the blood (Dumitrescu and Shields 2005). Higher estrogen exposure, in turn, can induce aberrant DNA methylation associated with breast carcinogenesis both in vivo and in vitro (Fernandez and Russo 2010). These observations suggest another possible mechanism of alcohol-induced carcinogenesis, at least in breast cancer.^2^

## Upper Aerodigestive Tract Cancer

Tobacco and alcohol are the major risk factors of upper aerodigestive tract cancers, or head and neck cancers, including cancers of the oral cavity, pharynx, larynx, and esophagus. Each year, about 125,000 new cases of upper aerodigestive tract cancers are diagnosed across Europe, and more than half of the patients die from the disease (Anantharaman et al. 2011).

Several studies have shown an association between alcohol and aberrant DNA methylation in head and neck cancers and that the degree of DNA hypomethylation is associated with alcohol use in these cancers. A study examining the DNA methylation profiles of 1,413 CpG loci from 773 genes in head and neck squamous cell carcinomas showed that significant associations existed between methylation profiles and alcohol consumption (Marsit et al. 2009). Other small-scale studies also have shown alcohol-associated promoter hypermethylation for several genes, including *E-cadherin* and *p16INKa* (Hasegawa et al. 2002), p15 (Chang et al. 2004), *MGMT* (Puri et al. 2005), *p14ARF* (Ishida et al. 2005), *SFRP1* (Marsit et al. 2006), and *Fusel 18* and *Septin 9* (Bennett et al. 2010). The genotype at the *C677T MTHFR* variant also plays a role in the risk of alcohol-related upper aerodigestive tract cancers. Thus, heavy-drinking individuals with the TT genotype have an increased risk of oral cancer compared with the CC genotype (Supic et al. 2011) but a decreased risk of esophageal cancer (Yang et al. 2005). Thus, the effect of the *MTHFR* polymorphism appears to differ substantially depending on the type of cancer.

## Conclusions and Outlook

Aberrant DNA methylation is a hallmark of cancer development, and many studies have shown its contribution to tumor initiation and progression. In fact, methylation patterns nowadays are used as markers for cancer detection, tumor prognosis, and prediction of treatment responses. At the moment, evidence suggests that alcohol use is associated with aberrant DNA methylation patterns in several types of cancer. However, most studies have relied on looking at individual genes or a limited number of CpG loci. Genome-wide DNA methylation analyses may yield comprehensive maps of DNA methylation changes in alcohol-associated carcinogenesis, which could be important for use in pharmacoepigenetics, serving as additional markers for cancer detection, prognosis, and treatment response. Furthermore, despite all the progress that has been made in elucidating how alcohol consumption might lead to altered DNA methylation patterns, the molecular mechanisms that lead to these alterations have to be better characterized so that effective therapies could be devised.

## Figures and Tables

**Table 1:** Abbreviations of Gene Names, Protein Names, and Other Molecules

**Abbreviation**	**Spelled-out Name**
**Genes**	
*APC-1A*	Adenomatous polyposis coli
*BRCA1*	Breast cancer 1, early onset
*CHRNA3*	Cholinergic re ceptor, nicotinic, alpha 3
*CDKN2A*	Cyclin-dependent kinase inhibitor 2A
*p14ARF*	Cyclin-dependent kinase inhibitor 2A, isoform 4
*CDKN2B, p15INK4b*	Cyclin-dependent kinase 4 inhibitor B
*CDKN2A, p16Ink4A*	Cyclin-dependent kinase inhibitor 2A
*ER-α*	Estrogen receptor
*GATA2*	GATA binding protein 2
*GSTP1*	Glutathione S-transferase pi 1
*HOX2A*	homeobox B5
*MGMT*	O-6-Methylguanine-DNA methyltransferase
*hMLH1*	MutL homolog 1
*RASSF1A*	Ras association (RalGDS/AF-6) domain family member 1
*RB*	Retinoblastoma
**Proteins**	
BHMT	Betaine homocysteine methyltransferase
DHFR	Dihydrofolate reductase
DNMTs	DNA methyltransferases
LINE family member L1, LINE-1	Long interspersed nucleotide element-1
MAT	Methionine adenosyltransferase
MTR	Methionine synthase
MTHFR	methylenetetrahydrofolate reductase
MT	Methyltransferase
SFRP1	Secreted frizzled-related protein 1
TS	Thymidylate synthase
**Other Compunds**	
DHF	Dihydrofolate
dTMP	Deoxythymidine monophosphate
dUMP	Deoxyuridine monophosphate
Hcy	Homocysteine
Met	Methionine
5,10-MTHF	5,10-Methylenetetrahydrofolate
5-MTHF	5-Methyltetrahydrofolate
SAH	S-adenosylhomocysteine
SAMe	S-adenosylmethionine
THF	Tetrahydrofolate

## Footnotes

1 The name of the variant indicates that the amino acid cysteine (abbreviated as C), which normally is found at position 677 in the MTHFR molecule, is replaced by a threonine (abbreviated as T). The names of other gene variants can be interpreted similarly.

2 A woman’s risk of breast cancer depends at least in part on her overall exposure to estrogen across her life span. Thus, women who begin menstruating earlier and reach menopause later have a higher cumulative estrogen exposure and, consequently, breast cancer risk than women with a shorter “menstrual life.” Similarly, postmenopausal alcoholic women have higher estrogen levels than women who are not alcoholic, increasing their overall estrogen exposure, which may in turn increase breast cancer risk.
